# The History of Methylprednisolone, Ascorbic Acid, Thiamine, and Heparin Protocol and I-MASK+ Ivermectin Protocol for COVID-19

**DOI:** 10.7759/cureus.12403

**Published:** 2020-12-31

**Authors:** Mika Turkia

**Affiliations:** 1 Qualitative Research, Independent Researcher, Helsinki, FIN

**Keywords:** covid-19, sars-cov-2, ivermectin, methylprednisolone, ascorbic acid, thiamine, heparin

## Abstract

An alliance of established experts on critical care, Front Line COVID-19 Critical Care Alliance (FLCCC), has published two protocols for treatment of COVID-19. The first one, methylprednisolone, ascorbic acid, thiamine, and heparin (MATH+), is intended for hospital and intensive care unit treatment of pulmonary phases of the disease. It is based on affordable, commonly available components: anti-inflammatory corticosteroids (methylprednisolone, "M"), high-dose vitamin C infusion (ascorbic acid, "A"), vitamin B1 (thiamine, "T"), anticoagulant heparin ("H"), antiparasitic agent ivermectin, and supplemental components ("+") including melatonin, vitamin D, elemental zinc, and magnesium.

The MATH+ protocol has received scarce attention due to the World Health Organization (WHO) advising against the use of corticosteroids in the beginning of the pandemic. In addition, randomized controlled clinical trials were required as a condition for adoption of the protocol. As the hospital mortality rate of MATH+ treated patients was approximately a quarter of the rate of patients receiving a standard of care, the authors of the protocol considered performing such trials unethical. Other parties have later performed clinical trials with corticosteroids and anticoagulants, which has led to a more widespread adoption of these components.

In October 2020, ivermectin was upgraded from an optional component to an essential component of the protocol. According to the authors, ivermectin is considered the first agent effective for both prophylaxis (prevention) of COVID-19 and for treatment of all phases of COVID-19 including outpatient treatment of the early symptomatic phase. Therefore, at the end of October 2020, a separate ivermectin-based I-MASK+ protocol for prophylaxis and early outpatient treatment of COVID-19 was published.

## Introduction

The first version of the methylprednisolone, ascorbic acid, thiamine, and heparin (MATH+) protocol created in January 2020 was based on corticosteroid hydrocortisone, vitamin C, and thiamine [1]. This protocol had been used for treatment of sepsis for several years. In January and February 2020, an anticoagulant was added to the protocol. Hydrocortisone was upgraded to methylprednisolone in April 2020. Ivermectin was added as an optional component in May 2020 and upgraded to an essential component in October 2020 (sources: protocol versions previously published at evms.edu/covidcare). 

In the beginning of the pandemic, corticosteroids were a controversial subject, possibly due to the phase-specific nature of COVID-19 not having been fully understood. The World Health Organization (WHO) had advised against the use of corticosteroids. This advisory was later proven to have been misguided but officially changed no earlier than September 2, 2020. Corticosteroids appear disadvantageous in the early symptomatic phase but necessary in the late pulmonary phase. Anticoagulants are another component that later became generally adopted.

In the first half of 2020 the hospital mortality rate of patients treated with MATH+ was 5%, in contrast to a standard of care rate of approximately 23% [1]. Due to this difference in mortality rates the authors of the MATH+ protocol did not consider performing randomized controlled trials ethical. As other parties refused to overlook this requirement, widespread adoption of the protocol did not occur. 

## Technical report

The Front Line COVID-19 Critical Care alliance perspective on COVID-19

According to the MATH+ protocol, COVID-19 progresses in phases. Different phases require slightly different, phase-specific treatment approaches [1]. Approximately 20 to 40 percent of the patients are estimated to be symptom-free. For symptomatic patients, there are four phases. The first phase is an approximately four-day symptomless incubation period. It is followed by an early symptomatic phase of approximately six days. The third phase is an early pulmonary phase of approximately three days. It is followed by a late pulmonary phase of approximately two weeks [1].

After a droplet or aerosol-mediated infection through airways or eyes, a symptomless incubation period follows, during which SARS-CoV-2 replicates predominantly in the nasal pharynx [2]. In this phase, antiviral agents such as quercetin or zinc may be beneficial [1].

Infectivity is the highest during the incubation period and in the beginning of the early symptomatic phase. After the early pulmonary phase the patient is no longer considered infective; yet tests based on detection of these virus particles may give a positive result. This is due to a fall in viral replication and viral load resulting to only non-replicable virus particles remaining in the body [3].

Since patients are typically not admitted to hospitals before the early pulmonary phase and because antiviral pharmaceuticals have been tested on these hospital patients, the antivirals have failed in trials. Patients do not die due to living viruses but due to a delayed, dysregulated immune response triggered by the non-replicable virus particles [4].

It is essential to begin controlling this inflammatory immune response immediately at the onset of symptoms before the inflammation causes organ damage that is difficult to repair. Therefore, an outpatient protocol should be applied immediately at the onset of mild symptoms.

After the early symptomatic phase, approximately one-fifth of the patients progress to the early pulmonary phase, which typically includes inflammation and organizing pneumonia [5]. Inflammation may progress through redundant cell-signaling pathways; therefore, blocking a single pathway with a single pharmaceutical is unlikely to inhibit progression of the disease. Therefore, the presented protocols utilize multiple agents to block multiple pathways and to gain synergistic effects. Suppressing the immune response in the early and late pulmonary phases is partially performed with corticosteroids. Of those, the alliance considers methylprednisolone especially suitable [1]. 

Another component for controlling the immune response is ascorbic acid. The rationale for using vitamin C infusion is that in the late pulmonary phase the vitamin C levels of the patients have been observed to reach levels corresponding to scurvy [6]. Vitamin C has broad-spectrum antiviral, antibacterial, and anti-inflammatory effects [7]. Methylprednisolone and vitamin C synergistically amplify each other's effects [8].

A central component of the MATH+ protocol is heparin (more specifically, enoxaparin belonging to the low molecular weight heparin family), which has also non-anticoagulant benefits [9]. In addition, MATH+ protocol utilizes thiamine which is often low in the elderly. Thiamine deficiency leads to diminished adenosine triphosphate (ATP) production in the cells and may contribute to mental confusion [10]. Melatonin may protect against multiorgan failure [11].

Ascorbic acid protocols in Shanghai and the United States

For more than a decade, vitamin C infusion had already been used in Shanghai, China, for severe illnesses requiring intensive care. In late 2019, a combination of high-dose vitamin C, heparin, anticoagulants, and antivirals was adopted as the official treatment protocol of COVID-19 in the Shanghai area [12].

The difference between the Shanghai protocol and the Marik protocol appears to be that the Shanghai recommendation was cautious toward corticosteroids and did not include thiamine, zinc, or quercetin. The national guideline of China published in English in March 2020 recommended heparin and corticosteroids, did not mention ascorbic acid but included anti-inflammatory traditional Chinese medicine preparations [13].

A randomized clinical trial of vitamin C for COVID-19 was started in China in February but due to the successful early containment of the epidemic, only 56 patients could be recruited [14]. Regardless, the treatment was shown to statistically significantly improve oxygenation (p = 0.01). On a 5% significance level (p = 0.05), groups differed with respect to IL-6 and bilirubin levels and, importantly, mortality of most severely ill patients. The hospital mortality rate of patients with a sequential organ failure assessment score equal to or larger than three was 22%, in contrast to 52% in the control group. If the study had not ended early due to lack of patients, the results would likely have been indisputable.

Ascorbic acid protocols in evidence-based medicine

As mentioned above, the MATH+ protocol had its origins in a vitamin C protocol developed for sepsis. The historical developments related to general adoption of this method promoted by another research group appear interesting. The adoption process seems to have been hindered by dogmatic adherence to formal requirements, an approach in which formalities seem to have mattered more than actual clinical outcomes or common sense. In the case of a decisive phase III sepsis trial [15], this meant funding body influenced selection of primary endpoints that in retrospect appeared poor and a methodological error that turned these endpoints statistically insignificant [16], resulting in the study being labeled negative and the method continuing to be largely ignored.

Disregarding what had been chosen for endpoints and looking at the actual data [15], it can be seen that during the vitamin C infusion, all-cause mortality rate was 5% in comparison to 23% in the control group and that the difference remained after discontinuation of the infusion until the end of the observation period of 28 days. Yet in the world of misunderstood or misapplied evidence-based medicine, this evidence was deemed irrelevant on a technicality. It should be noted that these kinds of research practices result not only in inferior science but also in huge societal inefficiencies. These historical biases have likely also hindered adoption of vitamin C for COVID-19. 

The role of ivermectin

Ivermectin has known antiviral effects on a broad range of RNA and DNA viruses. Significant effectiveness of ivermectin is seen in the early stages of infection. It has been suggested that ivermectin's mechanism of action may be predominantly anti-inflammatory or ionophoric [17]. A possible new mechanism of action has also been identified [18]. 

Despite the exact mechanism of action being unknown, trials continue to indicate an effect. In the end of October, Front Line COVID-19 Critical Care (FLCCC) alliance provided a review of ivermectin trials [19]. In one trial, ivermectin prevented appearance of symptoms in the outpatients' family members by a factor of seven (7% vs 58%) (NCT04422561). In a health personnel trial with ivermectin and iota carrageenan, infections were reduced from 11% to zero (NCT04425850). In another health personnel ivermectin prophylaxis trial, infections were reduced from 10% to 2% [19].

An ivermectin and doxycycline trial indicated shortened course of disease, reduction in the number of patients that remained persistently positive for reverse transcription polymerase chain reaction (RT-PCR) of COVID-19, and prevention of clinical deterioration (NCT04523831). Another trial with the same agents (NCT04591600) yielded similar results. 

In Peru, despite lack of randomized controlled trials at the time, ivermectin was officially adopted and nationally distributed since May 8, 2020. The idea seems to have emerged from successful grassroots experimentation in Iquitos. Epidemiological statistics indicate a declining trend in excess mortality [19]. Ivermectin has subsequently been distributed in many areas of Brazil, Bolivia, and Argentina. 

In the end of November, an anonymous group providing semi-automated meta-analyses published a meta-analysis of 21 ivermectin trials (ivmmeta.com). It indicated ivermectin's effectiveness for prophylaxis and treatment of all phases of COVID-19. A significant number of ivermectin trials are still ongoing or being planned [20].

## Discussion

Two of the main components, corticosteroids and heparin, of the MATH+ protocol presented in January to March 2020 have been generally adopted in the second half of 2020. Also thiamine is often being supplemented. Ascorbic acid remains unadopted. Considering that patients with severe disease have been shown to essentially suffer from scurvy, ascorbic acid has been shown safe and adopted as a standard of care in Shanghai area and has been used for hundreds of patients in two US hospitals with significant benefits on mortality, it is difficult to see a rationale behind non-adoption. 

Considering the roughly four-fold difference in mortality rates in the first half of 2020 [1], it seems unfortunate that the MATH+ protocol was largely ignored. It might be even more unfortunate to repeat the same mistake with ivermectin and continue exacerbating the societal costs of the pandemic by not reacting in time. The main interests with ivermectin are prophylaxis and outpatient treatment. Current data suggests significant benefits. As there are no concerns regarding safety or cost, it seems unadvisable to wait for more data. In an emergency situation, dogmatic adherence to extremely slow and inflexible evidence-based medicine practices may result in exactly the kind of harm it was intended to protect the society from.

## Conclusions

The MATH+ protocol correctly predicted the benefits of corticosteroids and anticoagulants, being ahead of its time in the first half of 2020. It has been continuously updated to reflect the current state of research. The adoption suffered due to a mismatch with WHO guidelines, the authors' foresight of the method being too efficient to ethically allow for a placebo-controlled study, and a related Chinese trial ending early due to lack of patients. 

Even though many of the essential parts of the MATH+ protocol have already been adopted in clinical practice, adopting the full protocol would likely still improve therapeutic efficacy in many inpatients. A recent addition, ivermectin, appears to be a cost-effective adjunct or alternative to vaccinations. Also the ivermectin recommendation was given before the publication of the majority of trial results. Adoption of ivermectin prophylaxis and early outpatient treatment might significantly reduce the need for lockdowns and other societal restrictions, easing also the financial burden of COVID-19. 
